# How Chef-Designed Plating Shapes Consumer Evaluations of a Dish in a Real Restaurant Setting

**DOI:** 10.3390/foods15173081

**Published:** 2026-08-31

**Authors:** Kosuke Motoki, Kohei Yamaguchi, Nozomi Koda, Hagino Oshiro, Ayane Horike, Rieko Moritoyo, Chika Takahashi, Tatsuya Noda, Haruka Tohara, Shin-ichi Ishikawa

**Affiliations:** 1 Faculty of Business and Commerce, Keio University, Tokyo 108-8345, Japan; 2 Department of Management, the University of Tokyo, Tokyo 113-0033, Japan; bigcastle@g.ecc.u-tokyo.ac.jp; 3 Department of Dysphagia Rehabilitation, Graduate School of Medical and Dental Sciences, Institute of Science Tokyo, Tokyo 113-8510, Japan; leaf126turtle@gmail.com (N.K.); ayanehorike55@gmail.com (A.H.); morito_yorie@yahoo.co.jp (R.M.); takahashi.dysph@gmail.com (C.T.); harukatohara@hotmail.com (H.T.); 4 PLUM KNOT Co., Ltd., Tokyo 104-0061, Japan; noda@nol.jp; 5 Department of Food Science and Business, Miyagi University, Sendai 982-0215, Japan; ishikawa@myu.ac.jp

**Keywords:** plate presentation, plating design, sustainable food, sustainable seafood, consumer evaluation, restaurant dining

## Abstract

Visual cues strongly influence consumer evaluations of food, but sauce presentation may also affect how diners interact with a dish during consumption. In a naturalistic restaurant field study, 57 participants completed a counterbalanced within-participant comparison of minimal and drizzle sauce presentations, with evaluations collected before and after tasting. An integrated 2 (presentation) × 2 (phase) × 2 (presentation order) repeated-measures analysis showed that, before tasting, minimal presentation was rated as healthier than drizzle presentation after adjustment for order (adjusted mean difference = 0.60, 95% CI [0.19, 1.00], *p* = 0.005; Holm- and FDR-adjusted *p* = 0.032). After tasting, minimal presentation received higher unadjusted ratings of intention to eat more and overall liking, but neither difference survived multiplicity correction. No presentation × phase interaction was significant. Because sauce placement also changed diners’ opportunity to regulate sauce use, post-consumption findings are interpreted as effects of presentation-and-consumption format rather than visual presentation alone. Thus, the clearest finding concerns pre-consumption healthiness inference, whereas the post-consumption results are smaller and exploratory.

## 1. Introduction

Eating is inherently a multisensory experience involving taste, smell, touch, and vision [1], yet vision plays a particularly important role among these senses [2,3]. Consumers inevitably rely on visual information before tasting food, as reflected in the popular phrase that diners “taste with their eyes first” [4]. Accordingly, the visual appearance of food often shapes consumers’ expectations and first impressions of a dish before tasting and can even influence perceptions during tasting. For example, color, shapes, forms, and visual elements in food itself [5], packaging [6], brand logos [7], and environmental cues [8] influence taste expectations, emotions, attitudes, and intentions, among others.

In restaurant contexts, these visual cues are largely determined by plate presentation—that is, how food is arranged and visually designed on the plate [9,10]. The visual presentation of dishes has long attracted attention not only among chefs and culinary practitioners but also among researchers interested in how plating shapes diners’ perceptions and experiences (see [11,12] for reviews). There has been a growing body of research examining how the visual appearance of a dish shapes consumer expectations and preferences regarding its taste, quality, and overall liking, among others [12,13,14,15,16,17,18,19,20]. Previous research has investigated the effects of plate size (small vs. large), plating arrangement (vertically stacked vs. horizontally spread) [21], esthetic features [13], neatness [19,20], and shape [22] on consumer evaluations. For example, research has shown that salads served on a large plate with a vertically stacked arrangement received the highest ratings for liking, compared to the other conditions [21].

Relatively little attention has been paid to plating styles created through sauces and condiments. In contemporary restaurant cuisine, chefs frequently use sauces not only for flavor but also as visual design elements on the plate. For example, sauces may be placed minimally and neatly on the plate or applied through drizzle patterns [14,23]. These arrangements may affect both visual impressions before tasting and the way diners apply and consume sauce after tasting. However, despite the prevalence of sauce-based design in contemporary restaurant practice, little is known about how such presentation-and-consumption formats shape expectations and post-consumption evaluations.

This issue may be particularly relevant in sustainability-oriented dining contexts, where underused ingredients or co-products may be incorporated into dishes. In the present study, both conditions used the same sturgeon-filet dish; sustainability was not manipulated. Sustainability is therefore treated as the culinary context of the experiment rather than as a causal factor explaining any presentation effect.

To address these issues, the present study examines evaluations of a sturgeon-filet dish presented using two sauce arrangements: minimal plating, in which sauces were placed separately on the plate, and drizzle sauce plating, in which sauces were distributed across the plate. Evaluations were measured both before and after tasting. The pre-consumption comparison primarily concerns visible presentation, whereas the post-consumption comparison may reflect both presentation and differences in sauce use.

Moreover, consumers’ evaluations of food may also change after tasting the dish. Prior research on novel and unfamiliar foods suggests that direct tasting experiences can reduce uncertainty and improve consumer acceptance [24,25]. Because consumers often form expectations based on visual cues before tasting, the actual eating experience may update these expectations and shape subsequent evaluations. Therefore, examining both pre-consumption expectations and post-consumption evaluations provides a more complete understanding of how visual presentation influences consumer responses during the dining experience.

We organize these questions using an expectancy–experience framework. Before tasting, sauce arrangement can operate through a visual-inference pathway by shaping impressions such as healthiness, simplicity, or apparent quantity. During consumption, however, the arrangement can also operate through a functional pathway because sauce location affects food coverage and the diner’s opportunity to regulate application. The present design can distinguish the pre-consumption visual contrast from the post-consumption presentation-and-serving configuration, but it cannot isolate the mechanisms within either pathway. Because the study was not preregistered and no primary outcome was specified in advance, the analyses are described as exploratory; the multiplicity-adjusted findings receive greatest interpretive weight.

Accordingly, the present research addresses three research questions. First, does chef-designed sauce presentation influence consumers’ expectations before tasting? Second, does the presentation-and-consumption format influence evaluations after tasting? Third, how do evaluations change from before to after tasting, and do these changes differ across formats after accounting for presentation order? Because no primary outcome was preregistered, the analyses are treated as exploratory and accompanied by multiplicity corrections and confidence intervals.

## 2. Materials and Methods

### 2.1. Participants

A total of 58 participants were recruited by convenience/snowball sampling through the researchers’ personal networks between February and March 2026. There were no specific eligibility criteria. No monetary compensation was provided; however, participants received a takeaway lunch box after completing the study. Participants had no formal relationship with the restaurant or researchers beyond study recruitment. One participant was excluded because they did not consume the food provided, leaving 57 participants. Among the 56 who reported age and gender, 32 identified as male and 24 as female; mean age was 33.43 years (SD = 7.61). The sample should not be considered representative of Japanese consumers or restaurant customers. The original a priori calculation used a two-tailed paired-samples *t* test and dz = 0.40 as a medium-effect planning benchmark, yielding a target of 52 at α = 0.05 and 80% power; it did not account for the later multi-outcome strategy.

### 2.2. Experimental Design

**Figure 1:**
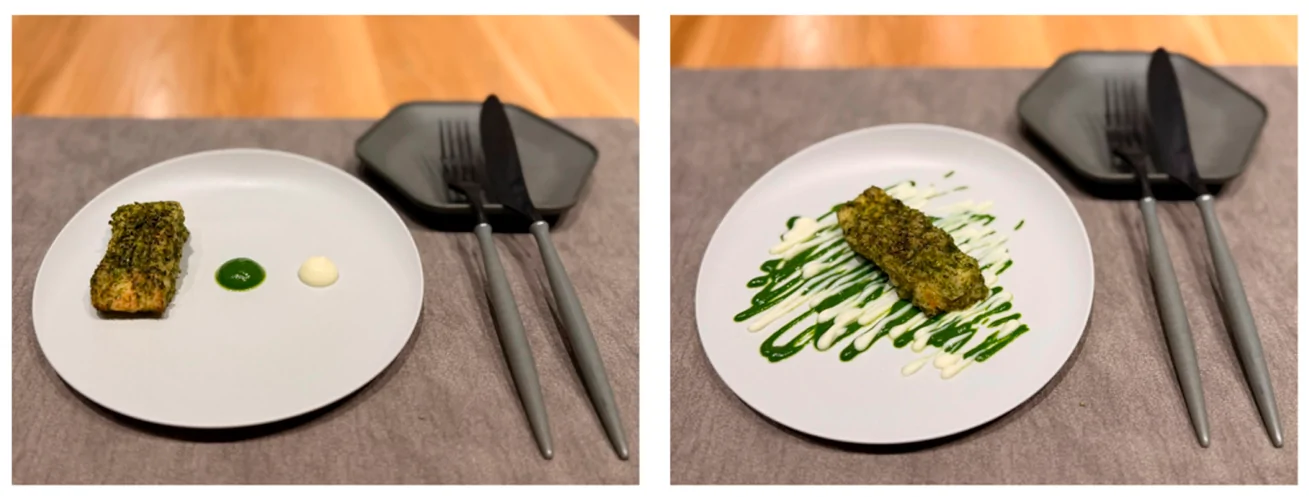
Example stimuli used in the experiment: minimal plating (**left**) and drizzle sauce presentation (**right**).

The study employed a within-participant design with two conditions: minimal plating and drizzle sauce presentation (Figure 1). In the minimal condition, sauces were placed separately on the plate; in the drizzle condition, sauces were distributed across the plate and around the food. Neutral labels are used because perceived artistic presentation did not differ between conditions. Sequence was not randomly assigned; the experimenters quasi-counterbalanced presentation order, yielding 29 drizzle-first and 28 minimal-first participants.

The dish used in this study was prepared using sturgeon filet in collaboration with a professional chef who has received a Michelin Green Star. Sturgeon aquaculture is primarily oriented toward caviar production, while meat is a quantitatively important co-product; fuller use of sturgeon meat and other co-products may improve resource efficiency [26,27]. We therefore describe the filet as less commercially prominent than caviar rather than claiming that the specific filet used here would otherwise have been discarded. Both conditions used the same dish and sauce types. Participants were not given information about sustainability or production methods.

### 2.3. Measures and Procedure

The study was conducted at 8go in Nihonbashi, Tokyo, Japan, within the Gastronomy Innovation Campus (GIC) Tokyo. Participants were told that they would evaluate two sequentially served sturgeon-fritter dishes, that there were no correct answers, and that they should report their own impressions. No hypothesis, sustainability information, or production-method information was disclosed. Exact food and sauce quantities, serving temperature, response duration, and actual sauce intake were not recorded. The second dish was served after participants had finished the first dish and completed its evaluation. Lighting and seating followed the restaurant’s usual conditions, and participants did not interact during the experiment. The experimenters quasi-counterbalanced the sequence without random assignment.

#### 2.3.1. Pre-Consumption Evaluation (Before Tasting)

For each presentation condition, participants evaluated the dish before tasting. The items were expected tastiness (“looks delicious”), expected healthiness (“is healthy”), intention to eat (“would like to try/eat it”), overall liking (“is favorable overall”), and perceived artistic presentation (“the plating is artistic”).

Participants indicated WTP from 0 to 3000 JPY for a full six-piece serving, after being shown a one-piece tasting portion. They also reported intention to visit the restaurant, word-of-mouth intention, willingness to try the dish if served with a sustainable ingredient, and appropriateness for inclusive dining. All non-WTP outcomes were assessed as single items using a seven-point agreement response format with verbal anchors at 1 = strongly disagree (まったくそう思わない), 4 = neither agree nor disagree (どちらでもない), and 7 = strongly agree (非常にそう思う); points 2, 3, 5, and 6 were numbered but verbally unlabeled. These measures were not multi-item Likert scales. Although tastiness and overall liking concern hedonic evaluations, the response format was also not the conventional nine-point hedonic scale ranging from “dislike extremely” to “like extremely” [28]. A seven-category format was selected to use a common response structure across constructs, retain a neutral midpoint, provide adequate discrimination, and minimize respondent burden in a restaurant field setting. Exact Japanese wording and English translations are provided in Supplementary Table S1.

#### 2.3.2. Post-Consumption Evaluation (After Tasting)

After tasting each presentation, participants rated perceived tastiness (“is delicious”), perceived healthiness (“is healthy”), intention to eat more (“would like to eat more”), overall liking (“is favorable overall”), WTP, visit intention, and word-of-mouth intention. The pre-consumption intention item and post-consumption intention-to-eat-more item were related but not identical; phase-related analyses are therefore interpreted cautiously.

This evaluation procedure was conducted for both presentation conditions. Finally, participants reported age and gender. Hunger was measured before each dish using a seven-point scale (1 = not at all hungry, 7 = very hungry). Evaluation measures such as tastiness, liking, and WTP were selected because similar attributes have been used in prior research on plate presentation and food esthetics [5,14,20]. Visit and word-of-mouth intentions were included as common restaurant outcomes [29,30]. Sustainability-related and inclusive-dining measures were exploratory.

### 2.4. Statistical Analyses

Analyses used a 2 (presentation: minimal vs. drizzle; within participant) × 2 (phase: pre- vs. post-consumption; within participant) × 2 (presentation order: drizzle-first vs. minimal-first; between participant) repeated-measures framework. Participant-level contrasts represented presentation, phase, presentation × phase, and interactions with order. Order-adjusted contrasts equally weighted the two sequences. Paired condition contrasts were also reported separately for pre- and post-consumption outcomes. Holm and Benjamini–Hochberg FDR corrections were applied within pre-consumption, post-consumption, and integrated-model effect families. Mean differences, 95% confidence intervals, and Cohen’s dz with participant-bootstrap 95% confidence intervals (30,000 resamples) are reported. WTP was additionally evaluated using Wilcoxon signed-rank, paired sign-flip permutation, and log-transformed paired tests. Hunger did not differ between conditions, *t*(56) = −0.24, *p* = 0.808. Because the seven-point items are ordinal single-item ratings, the *t*-based analyses were supplemented with Wilcoxon signed-rank sensitivity analyses. Parametric procedures are generally robust for Likert-type ordinal responses under many conditions [31], but the nonparametric analyses avoid relying on equal category intervals. The sensitivity analyses produced the same substantive pattern as the primary analyses. For clarity, Table 1 and Table 2 report unadjusted paired-samples tests (df = 56), whereas the corresponding order-adjusted contrasts use df = 55; small differences in estimates, confidence intervals, t values, and *p* values therefore reflect different analyses rather than inconsistent calculations. Table 3 reports order-adjusted presentation × phase interaction tests (df = 55). All analyses were conducted using R version 4.1.3 (R Foundation for Statistical Computing, Vienna, Austria).

## 3. Results

### 3.1. Pre-Consumption Evaluation (Before Tasting)

**Figure 2:**
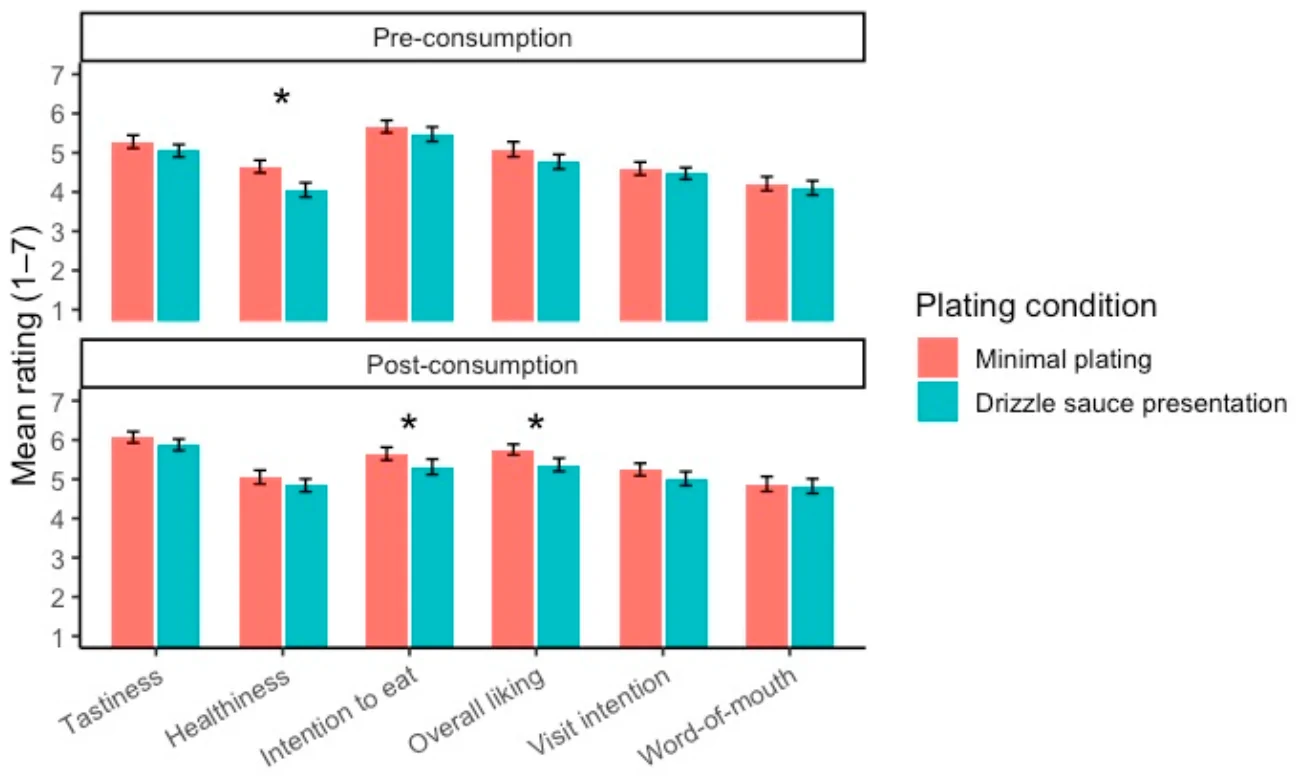
Descriptive mean ratings by plating condition before and after tasting. Note. Error bars represent ±1 standard error. Asterisks indicate unadjusted paired comparisons at *p* < 0.05; post-consumption intention and liking differences did not survive multiplicity correction.

Before tasting, minimal plating was rated as healthier than drizzle presentation. The order-adjusted mean difference was 0.60, 95% CI [0.19, 1.00], *t*(55) = 2.95, *p* = 0.005; this remained significant after Holm and FDR correction across repeated outcomes (adjusted *p* = 0.032). In the full 10-outcome pre-consumption family, the paired contrast also survived correction (raw *p* = 0.004; Holm/FDR-adjusted *p* = 0.042), dz = 0.39, bootstrap 95% CI [0.14, 0.69]. No other pre-consumption contrast was significant after correction. Perceived artistic presentation did not differ between conditions. The ordinal sensitivity analysis confirmed the healthiness difference, Wilcoxon *p* = 0.005; Holm- and FDR-adjusted *p* = 0.048. Descriptive mean ratings by plating condition before and after tasting are shown in Figure 2.

### 3.2. Post-Consumption Evaluation (After Tasting)

After tasting, Table 2 reports the unadjusted paired-samples contrasts (df = 56), whereas the corresponding order-adjusted estimates use df = 55. For intention to eat more, the unadjusted estimate was 0.33, 95% CI [0.02, 0.64], *t*(56) = 2.15, *p* = 0.036, and the order-adjusted estimate was 0.33, 95% CI [0.02, 0.65], *t*(55) = 2.13, *p* = 0.038. For overall liking, the unadjusted estimate was 0.39, 95% CI [0.09, 0.68], *t*(56) = 2.65, *p* = 0.010, and the order-adjusted estimate was 0.38, 95% CI [0.09, 0.67], *t*(55) = 2.64, *p* = 0.011. Neither order-adjusted difference survived correction across the seven post-consumption outcomes (intention: Holm *p* = 0.227, FDR q = 0.133; liking: Holm/FDR = 0.075). These multiplicity-adjusted *p* values are from the order-adjusted contrasts; Table 2 reports the corresponding adjusted *p* values for the unadjusted paired-samples tests. Paired-test effect sizes were small: dz = 0.28, bootstrap 95% CI [0.04, 0.53], for intention and dz = 0.35, bootstrap 95% CI [0.12, 0.58], for liking. Perceived tastiness was non-significant in both analyses (order-adjusted raw *p* = 0.063; unadjusted paired-samples test in Table 2: *t*(56) = 1.90, *p* = 0.062); no “marginal trend” interpretation is made. Wilcoxon sensitivity analyses similarly yielded raw *p* = 0.047 for intention to eat more and *p* = 0.009 for overall liking; neither survived Holm correction across the six post-consumption ordinal outcomes (adjusted *p* = 0.233 and 0.052, respectively). WTP was also non-significant under every robustness analysis. Before tasting, the median was JPY 1200 in both conditions (minimal range 200–3000; drizzle range 100–2600), and after tasting the medians were JPY 1200 and 1400 (ranges 200–2800 and 150–2800). Distribution skewness was 0.74 and 0.43 before tasting and 0.43 and 0.05 after tasting. Wilcoxon *p* values were 0.763 before tasting and 0.379 after tasting; sign-flip and log-WTP tests likewise yielded no evidence of a condition difference.

### 3.3. Changes in Evaluations After Tasting (Post-Consumption Minus Pre-Consumption)

**Figure 3:**
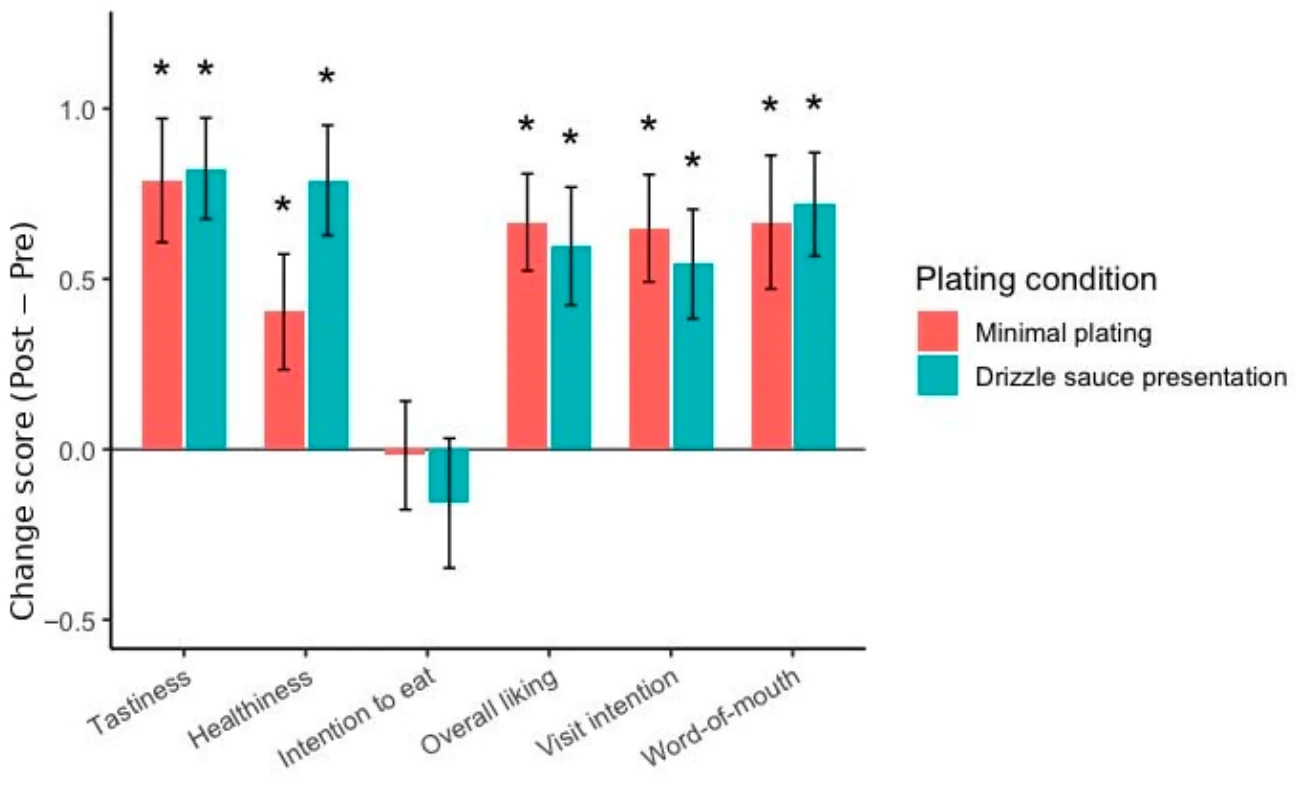
Descriptive changes in dish evaluations after tasting across plating conditions. Note. Error bars represent ±1 standard error. Asterisks indicate unadjusted one-sample comparisons with zero; presentation × phase interactions were non-significant.

Presentation × phase interactions were non-significant for all seven repeated outcomes after adjustment for order (all raw ps ≥ 0.063; Holm-adjusted ps ≥ 0.440). Thus, pre-to-post change did not differ reliably between presentations. The presentation × phase × order interaction for intention had raw *p* = 0.024 but did not survive correction (Holm/FDR = 0.170). This sequence-related pattern is reported transparently but interpreted cautiously, particularly because the pre- and post-consumption intention items were not identical. Descriptive change scores are presented in Figure 3 and complete inferential results in Table 3.

## 4. General Discussion

### 4.1. Summary of Findings

The present study compared minimal and drizzle sauce presentations in a real restaurant. Before tasting, minimal presentation produced a robust increase in perceived healthiness. After tasting, minimal presentation showed small unadjusted advantages for intention to eat more and overall liking, but these differences did not survive multiplicity correction. Most outcomes did not differ, and no presentation × phase interaction was significant. The strongest conclusion therefore concerns pre-consumption healthiness inference; post-consumption findings are exploratory and may reflect sauce use as well as visual arrangement.

### 4.2. Theoretical Contribution

The general proposition that visual presentation affects food expectations is already well established; the incremental contribution here is narrower. The study examines sauce arrangement, an understudied element that is simultaneously visual and consumable, using the same dish, chef, diners, and restaurant before and after actual tasting [9]. Only the pre-consumption healthiness inference remained reliable after multiplicity correction, whereas no post-consumption difference did. This boundary condition suggests that sauce configuration may shape a specific expectation without reliably altering experienced evaluations. Sauces and condiments contribute simultaneously to visual composition, flavor delivery, food coverage, and diner control. Separate placement may increase whitespace and permit self-regulated application, whereas drizzle presentation increases distributed coverage. The present design cannot isolate these pathways. Future studies should independently manipulate decorative pattern, total sauce quantity, plate coverage, and freedom of application, while measuring perceived control and weighing plates before and after consumption. This would distinguish expectation-related visual effects from dose, flavor-exposure, and consumption-related effects.

Second, the study contributes methodologically by examining plating effects in a real restaurant environment in collaboration with a professional chef. While much of the existing research on food presentation has been conducted using images or controlled laboratory settings, only a limited number of studies have investigated plating effects in real restaurant contexts involving actual eating experiences [14,15]. The present study extends this line of work by examining sauce presentation in a working restaurant setting. By studying consumer responses to dishes prepared and served by a professional chef in an actual dining environment, the research enhances the ecological validity of plating research and provides insights into how visual presentation operates in realistic culinary contexts. This approach also aligns with recent methodological work mapping the manipulable dimensions of food, service, and ambience in real restaurant experiments [32].

Third, the study was situated in sustainability-oriented gastronomy, a context relevant to work on novel foods and sustainable seafood [33,34]. However, sustainability was not manipulated or made salient in the experimental instructions. The findings therefore do not show that plating promotes acceptance specifically because a dish is sustainable; rather, they provide evidence from one sustainability-oriented restaurant context.

### 4.3. Plating Simplicity and Perceived Healthiness

One possible explanation for the pre-consumption healthiness result is visual simplicity. Prior work indicates that lower visual density and simple design can increase healthiness or naturalness inferences [35,36]. However, simplicity, naturalness, expected calories, whitespace, apparent sauce quantity, and visual density were not measured here. These possibilities should therefore be treated as hypotheses for future research rather than mechanisms demonstrated by the current data.

### 4.4. Plating Style and Post-Consumption Evaluations

The small post-consumption differences may relate to the greater consumption flexibility of the minimal format. Separately placed sauce allowed diners to regulate application, whereas drizzle presentation distributed sauce across the dish. Customization research suggests that control can enhance evaluation [37,38], but perceived control, sauce preference, and actual intake were not measured, and the observed differences did not survive multiplicity correction. Consumer control is therefore only a possible explanation.

### 4.5. Post-Consumption Improvements in Evaluations

Several evaluations increased after tasting, consistent with prior research showing that direct exposure can improve responses to unfamiliar foods [24,25]. Nevertheless, prior familiarity with sturgeon was not measured, and presentation × phase interactions were non-significant. The increases could reflect sensory quality, expectation updating, repeated measurement, or other features of the restaurant experience; they cannot be attributed specifically to reduced unfamiliarity.

### 4.6. Practical Implications

The robust pre-consumption healthiness result suggests that minimal sauce presentation may communicate a healthier impression. This implication should be limited to perceived healthiness rather than actual nutritional quality. Prior work indicates growing practitioner interest in healthier and more sustainable food systems [39].

The post-consumption findings should be interpreted more cautiously. Presenting sauce separately may provide flexibility, but the effects were small, did not survive multiplicity correction, and may depend on unmeasured sauce use. Future experiments should directly measure perceived control and intake.

Tasting may update evaluations of unfamiliar ingredients, but this study did not measure actual purchase, repeat consumption, or longitudinal acceptance. Restaurants and producers should therefore view these results as preliminary rather than as evidence of sustained adoption.

### 4.7. Limitations and Future Research

Despite its contributions, this study has several limitations. It examined one dish in one restaurant and used a convenience/snowball sample recruited through researchers’ networks. The sample was not representative, and the original power calculation addressed one paired comparison rather than the full multi-outcome strategy. Generalization should therefore be limited to this sample, dish, restaurant, and cultural setting.

Second, the manipulation changed both the presentation and consumption format. Sauce was distributed across the dish in the drizzle condition but placed separately in the minimal condition. Exact quantities, serving temperature, response duration, and actual intake were not recorded. Consequently, visual appearance, sauce exposure, control, sauce-to-food contact, and intake may have differed; post-consumption effects cannot be uniquely attributed to visual plating.

Third, the study focused on one sauce-based manipulation [23]. Perceived artistic presentation did not differ between conditions, so drizzle presentation should not be interpreted as empirically more artistic. Future research should separately measure visual density, whitespace, simplicity, naturalness, apparent sauce quantity, control, and artistry, while considering broader design principles such as artistic presentation and balance [14,40].

A further limitation concerns the measurement format. Each construct was assessed with a single seven-point agreement item rather than a validated multi-item instrument, and liking was not assessed with the conventional nine-point hedonic scale. The single-item format reduced burden between servings in a working restaurant but did not permit assessment of internal consistency and treated hedonic constructs in an agreement-response format. We addressed the ordinal character of the ratings through Wilcoxon sensitivity analyses, which supported the same substantive conclusions, but a conventional hedonic scale could yield a different response distribution. Future work should combine validated hedonic measurement with just-about-right ratings of sauce quantity and direct measures of perceived coverage, control, and actual intake.

Another limitation is possible demand characteristics because all participants saw and consumed both versions of the dish. Instructions identified only the first and second sturgeon-fritter dishes, stated that there were no correct answers, and did not disclose a hypothesis or sustainability information; however, no formal blinding or cover story was used. The generally favorable pre-consumption ratings may also have restricted the effect of presentation [20,41].

## 5. Conclusions

Minimal sauce presentation produced a robust pre-consumption increase in perceived healthiness, confirmed by an ordinal sensitivity analysis. Post-consumption differences were small, did not survive multiplicity correction, and cannot be separated from differences in sauce distribution, exposure, and diner control. The contribution is therefore bounded: sauce configuration influenced one expectation before tasting but did not reliably alter experienced evaluations. Sustainability was the culinary context rather than a manipulated cause. Conclusions are limited to one dish, restaurant, convenience sample, and cultural setting; confirmatory replication should preregister outcomes, use validated hedonic measures, standardize sauce quantity, and record actual intake.

## Figures and Tables

**Table 1:** Descriptive statistics and paired-samples tests for pre-consumption evaluations.

Ratings	Minimal M (SD)	Drizzle Sauce M (SD)	Difference [95% CI]	*t* (df)	*p*/Holm/FDR	dz [95% CI]
Expected tastiness	5.28 (1.26)	5.05 (1.20)	0.23 [−0.14, 0.60]	1.24 (56)	0.220/1.000/0.734	0.16 [−0.09, 0.45]
Expected healthiness	4.65 (1.22)	4.05 (1.39)	0.60 [0.20, 1.00]	2.98 (56)	0.004/0.042/0.042	0.39 [0.14, 0.69]
Intention to eat	5.67 (1.20)	5.47 (1.39)	0.19 [−0.21, 0.60]	0.96 (56)	0.341/1.000/0.783	0.13 [−0.13, 0.38]
Overall liking	5.09 (1.44)	4.77 (1.40)	0.32 [−0.12, 0.75]	1.46 (56)	0.149/1.000/0.734	0.19 [−0.07, 0.46]
Perceived artistic presentation of the plating	4.67 (1.53)	4.60 (1.62)	0.07 [−0.54, 0.68]	0.23 (56)	0.818/1.000/0.818	0.03 [−0.23, 0.30]
WTP for the dish	1206.84 (618.61)	1168.42 (556.60)	38.42 [−78.54, 155.39]	0.66 (56)	0.513/1.000/0.783	0.09 [−0.19, 0.33]
Intention to visit the restaurant	4.60 (1.27)	4.47 (1.12)	0.12 [−0.24, 0.48]	0.69 (56)	0.495/1.000/0.783	0.09 [−0.17, 0.36]
Word-of-mouth intention	4.21 (1.36)	4.11 (1.37)	0.11 [−0.33, 0.54]	0.49 (56)	0.626/1.000/0.783	0.06 [−0.20, 0.33]
Willingness to try the dish if it were served with sustainable ingredients	5.02 (1.61)	4.91 (1.79)	0.11 [−0.27, 0.48]	0.56 (56)	0.579/1.000/0.783	0.07 [−0.20, 0.32]
Perceptions of whether the plating would be appropriate for inclusive dining	3.79 (1.18)	3.84 (1.46)	−0.05 [−0.42, 0.31]	−0.29 (56)	0.773/1.000/0.818	−0.04 [−0.29, 0.24]

Note. Differences are minimal minus drizzle. Raw, Holm-adjusted, and FDR-adjusted *p* values are shown. Cohen’s dz includes participant-bootstrap 95% CIs. Seven-point items used agreement anchors at 1, 4, and 7; WTP was 0–3000 JPY. Values in this table are unadjusted paired-samples tests (df = 56); order-adjusted contrasts reported in the text use df = 55.

**Table 2:** Descriptive statistics and paired-samples *t* tests for post-consumption evaluations.

Ratings	Minimal M (SD)	Drizzle Sauce M (SD)	Difference [95% CI]	*t* (df)	*p*/Holm/FDR	dz [95% CI]
Perceived tastiness	6.07 (1.10)	5.88 (1.10)	0.19 [−0.01, 0.40]	1.90 (56)	0.062/0.312/0.146	0.25 [0.00, 0.49]
Perceived healthiness	5.05 (1.33)	4.84 (1.24)	0.21 [−0.18, 0.60]	1.07 (56)	0.289/0.677/0.337	0.14 [−0.12, 0.42]
Intention to eat more	5.65 (1.25)	5.32 (1.48)	0.33 [0.02, 0.64]	2.15 (56)	0.036/0.215/0.125	0.28 [0.04, 0.53]
Overall liking	5.75 (1.02)	5.37 (1.26)	0.39 [0.09, 0.68]	2.65 (56)	0.010/0.072/0.072	0.35 [0.12, 0.58]
WTP for the dish	1294.04 (600.83)	1346.67 (587.23)	−52.63 [−138.69, 33.43]	−1.23 (56)	0.226/0.677/0.316	−0.16 [−0.39, 0.10]
Intention to visit the restaurant	5.25 (1.20)	5.02 (1.34)	0.23 [−0.05, 0.50]	1.66 (56)	0.102/0.408/0.178	0.22 [−0.04, 0.49]
Word-of-mouth intention	4.88 (1.44)	4.82 (1.40)	0.05 [−0.23, 0.34]	0.37 (56)	0.713/0.713/0.713	0.05 [−0.22, 0.32]

Note. Differences are minimal minus drizzle. Raw, Holm-adjusted, and FDR-adjusted *p* values are shown. Cohen’s dz includes participant-bootstrap 95% CIs. Seven-point items used agreement anchors at 1, 4, and 7; WTP was 0–3000 JPY. Values in this table are unadjusted paired-samples tests (df = 56); order-adjusted contrasts reported in the text use df = 55.

**Table 3:** Changes in evaluations after tasting (post-consumption minus pre-consumption).

Ratings	ΔMinimal M (SD)	ΔDrizzle M (SD)	Adjusted Interaction [95% CI]	*t* (df)	*p*/Holm/FDR
Perceived tastiness	0.79 (1.37)	0.82 (1.12)	−0.03 [−0.42, 0.35]	−0.17 (55)	0.866/1.000/0.866
Perceived healthiness	0.40 (1.28)	0.79 (1.22)	−0.39 [−0.80, 0.02]	−1.90 (55)	0.063/0.440/0.283
Overall liking	0.67 (1.07)	0.60 (1.31)	0.07 [−0.32, 0.46]	0.36 (55)	0.721/1.000/0.866
Intention to eat more	−0.02 (1.20)	−0.16 (1.44)	0.15 [−0.25, 0.55]	0.75 (55)	0.457/1.000/0.866
Willingness to pay (JPY)	87.19 (339.68)	178.25 (349.71)	−92.17 [−196.08, 11.73]	−1.78 (55)	0.081/0.486/0.283
Intention to visit the restaurant	0.65 (1.19)	0.54 (1.21)	0.10 [−0.23, 0.44]	0.62 (55)	0.537/1.000/0.866
Word-of-mouth intention	0.67 (1.48)	0.72 (1.15)	−0.06 [−0.47, 0.35]	−0.28 (55)	0.777/1.000/0.866

Note. Δ = post minus pre. SDs are participant-level change-score SDs. The interaction is (minimal post − pre) − (drizzle post − pre), adjusted for presentation order. Raw, Holm-adjusted, and FDR-adjusted *p* values are shown.

## Data Availability

The data presented in this study are available on request from the corresponding author.

## Supplementary Materials

The following supporting information can be downloaded at: https://www.mdpi.com/article/10.3390/foods15173081/s1, Table S1. Exact item wording and response formats.

## References

[1] Spence C. (2015). Multisensory flavor perception. Cell.

[2] Spence C., Okajima K., Cheok A.D., Petit O., Michel C. (2016). Eating with our eyes: From visual hunger to digital satiation. Brain Cogn..

[3] Spence C., Motoki K., Petit O. (2022). Factors influencing the visual deliciousness / eye-appeal of food. Food Qual. Prefer..

[4] van der Laan L.N., de Ridder D.T.D., Viergever M.A., Smeets P.A.M. (2011). The first taste is always with the eyes: A meta-analysis on the neural correlates of processing visual food cues. NeuroImage.

[5] Hagen L. (2021). Pretty healthy food: How and when aesthetics enhance perceived healthiness. J. Mark..

[6] Spence C., Van Doorn G. (2022). Visual communication via the design of food and beverage packaging. Cogn. Res. Princ. Implic..

[7] Motoki K., Velasco C. (2021). Taste-shape correspondences in context. Food Qual. Prefer..

[8] Motoki K., Takahashi A., Spence C. (2021). Tasting atmospherics: Taste associations with colour parameters of coffee shop interiors. Food Qual. Prefer..

[9] İşeri C., Ekincek S. (2025). Plating the design: A systematic review of plate presentation in gastronomy. Int. J. Gastron. Food Sci..

[10] Spence C. (2019). Reading the plate. Int. J. Gastron. Food Sci..

[11] Deroy O., Michel C., Piqueras-Fiszman B., Spence C. (2014). The plating manifesto (I): From decoration to creation. Flavour.

[12] Spence C., Piqueras-Fiszman B., Michel C., Deroy O. (2014). Plating manifesto (II): The art and science of plating. Flavour.

[13] Liu M., Ji S., Jiang B., Huang J. (2023). Plating for health: A cross-cultural study of the influence of aesthetics characteristics on food evaluation. Int. J. Gastron. Food Sci..

[14] Michel C., Velasco C., Gatti E., Spence C. (2014). A taste of Kandinsky: Assessing the influence of the artistic visual presentation of food on the dining experience. Flavour.

[15] Michel C., Velasco C., Fraemohs P., Spence C. (2015). Studying the impact of plating on ratings of the food served in a naturalistic dining context. Appetite.

[16] Michel C., Woods A.T., Neuhäuser M., Landgraf A., Spence C. (2015). Rotating plates: Online study demonstrates the importance of orientation in the plating of food. Food Qual. Prefer..

[17] Rowley J., Spence C. (2018). Does the visual composition of a dish influence the perception of portion size and hedonic preference?. Appetite.

[18] Velasco C., Veflen N. (2021). Aesthetic plating and motivation in context. Int. J. Gastron. Food Sci..

[19] Zellner D.A., Siemers E., Teran V., Conroy R., Lankford M., Agrafiotis A., Ambrose L., Locher P. (2011). Neatness counts. How plating affects liking for the taste of food. Appetite.

[20] Zellner D.A., Loss C.R., Zearfoss J., Remolina S. (2014). It tastes as good as it looks. The effect of food presentation on liking for the flavor of food. Appetite.

[21] Cobo M.I.S., Jager G., Ioannou O., de Graaf C., Zandstra E.H. (2025). Plate size or plating? Effects of visual food presentation on liking, appetite, and food-evoked emotions in online and real-life contexts. Food Qual. Prefer..

[22] Fairhurst M.T., Pritchard D., Ospina D., Deroy O. (2015). Bouba-Kiki in the plate: Combining crossmodal correspondences to change flavour experience. Flavour.

[23] Kimura A., Yamaguchi K., Tohara H., Sato Y., Sawada N., Nakagawa Y., Matsuda Y., Inoue M., Tamaki K. (2019). Addition of sauce enhances finger-snack intake among Japanese elderly people with dementia. Clin. Interv. Aging.

[24] Hartmann C., Siegrist M. (2016). Becoming an insectivore: Results of an experiment. Food Qual. Prefer..

[25] Chong M., Leung A., Fernandez T.M. (2024). On-site sensory experience boosts acceptance of cultivated chicken. Future Foods.

[26] Bronzi P., Chebanov M., Michaels J.T., Wei Q., Rosenthal H., Gessner J. (2019). Sturgeon meat and caviar production: Global update 2017. J. Appl. Ichthyol..

[27] Dudu A.S., Georgescu S.E. (2024). Exploring the multifaceted potential of endangered sturgeon: Caviar, meat and by-product benefits. Animals.

[28] Lim J. (2011). Hedonic scaling: A review of methods and theory. Food Qual. Prefer..

[29] Daries N., Cristobal-Fransi E., Sánchez-García J., Marine-Roig E. (2024). Customers’ behavioral intentions when visiting upscale restaurants: Enjoying the experience or posturing?. Int. J. Gastron. Food Sci..

[30] Öğretmenoğlu M., Akın G., Huang T.-Y.T., Kızılırmak İ. (2026). Effect of slow food experience on WoM: The moderating role of food neophilia and the mediating role of eudaimonic and hedonic well-being. Int. J. Gastron. Food Sci..

[31] Norman G. (2010). Likert scales, levels of measurement and the “laws” of statistics. Adv. Health Sci. Educ..

[32] Motoki K., Yamaguchi K., Velasco C. (2026). Designing real restaurant experiments: Mapping manipulation space across food, service, and ambience. Int. J. Gastron. Food Sci..

[33] Motoki K., Park J., Spence C., Velasco C. (2022). Contextual acceptance of novel and unfamiliar foods: Insects, cultured meat, plant-based meat alternatives, and 3D printed foods. Food Qual. Prefer..

[34] Roheim C.A., Bush S.R., Asche F., Sanchirico J.N., Uchida H. (2018). Evolution and future of the sustainable seafood market. Nat. Sustain..

[35] Huang Z., Wang L. (2026). Dense is calorie-rich: The effect of visual density in food packaging on calorie estimation. Psychol. Mark..

[36] Ton L.A.N., Smith R.K., Sevilla J. (2024). Symbolically simple: How simple packaging design influences willingness to pay for consumable products. J. Mark..

[37] Puligadda S., Grewal R., Rangaswamy A., Kardes F.R. (2010). The role of idiosyncratic attribute evaluation in mass customization. J. Consum. Psychol..

[38] Yoo J., Park M. (2016). The effects of e-mass customization on consumer perceived value, satisfaction, and loyalty toward luxury brands. J. Bus. Res..

[39] Bertoldo J., Hsu R., Reid T., Righter A., Wolfson J.A. (2022). Attitudes and beliefs about how chefs can promote nutrition and sustainable food systems among students at a US culinary school. Public Health Nutr..

[40] Velasco C., Michel C., Woods A.T., Spence C. (2016). On the importance of balance to aesthetic plating. Int. J. Gastron. Food Sci..

[41] Youssef J., Spence C. (2021). Introducing diners to the range of experiences in creative Mexican cuisine, including the consumption of insects. Int. J. Gastron. Food Sci..

