# Supplementary material for: How Chef-Designed Plating Shapes Consumer Evaluations of a Dish in a Real Restaurant Setting
Source: Foods. 2026 Aug 31;15(17):3081. doi: 10.3390/foods15173081 (PMC13565332; doi:10.3390/foods15173081)
Supplement: Supplementary file 1 [file foods-15-03081-s001.zip › foods-4495440-supplementary.pdf]

Supplementary Table S1. Exact item wording and response formats

The Japanese wording is reproduced from the original questionnaire screenshots supplied by the authors. English wording is a translation for reporting purposes. Unless otherwise stated, the scale was 1 = まったくそう思わない (strongly disagree), 4 = どちらでもない (neither agree nor disagree), and 7 = 非常にそう思う (strongly agree); intermediate points were numbered only.

Phase	Construct	Japanese wording	English translation	Response format
Pre	Expected tastiness	美味しそう	Looks delicious	1–7 agreement
Pre	Expected healthiness	健康的である	Is healthy	1–7 agreement
Pre	Intention to eat	食べてみたい	I would like to try/eat it	1–7 agreement
Pre	Overall liking	全体的に好ましい	It is favorable overall	1–7 agreement
Pre	Artistic presentation	盛り付けが芸術的だ	The plating is artistic	1–7 agreement
Pre	WTP	提供されている料理について、支払いたい金額（0〜3000 円）を回答してください。今回は 1 ピースでの提供ですが、一人前だと 6 ピースあります。それを考慮して一人前の支払いたい金額を回答してください。	Please report the amount you would be willing to pay (JPY 0–3000). Although one piece was served, a full portion contains six pieces; please estimate the price for one full portion.	Numeric, JPY 0–3000
Pre	Visit intention	この料理が提供されているレストランを訪れたい	I would like to visit a restaurant where this dish is served	1–7 agreement
Pre	Word-of-mouth intention	この料理について友人・知人に教えたい	I would like to tell friends or acquaintances about this dish	1–7 agreement
Pre	Sustainable-ingredient trial intention	サステナブルな食材がこの料理の盛り付けで提供されていたら、食べてみたい	If a sustainable ingredient were served with this plating, I would like to try it	1–7 agreement; exploratory
Pre	Inclusive dining	この料理の盛り付けはインクルーシブな食事（例：加齢や障害で噛む力・飲み込む力が弱い人でも食べられる）に相応しい	This plating is suitable for inclusive dining (e.g., for people with reduced chewing or swallowing ability due to aging or disability)	1–7 agreement; exploratory
Post	Perceived tastiness	美味しい	Is delicious	1–7 agreement
Post	Perceived healthiness	健康的だ	Is healthy	1–7 agreement
Post	Intention to eat more	より食べてみたい	I would like to eat more	1–7 agreement
Post	Overall liking	全体的に好ましい	It is favorable overall	1–7 agreement
Post	WTP	提供されている料理を食べてみて、支払いたい金額（0〜3000 円）を回答してください。今回は 1 ピースでの提供ですが、一人前だと 6 ピースあります。それを考慮して一人前の支払いたい金額を回答してください。	After tasting the dish, please report the amount you would be willing to pay (JPY 0–3000) for a full six-piece portion.	Numeric, JPY 0–3000
Post	Visit intention	この料理が提供されているレストランを訪れたい	I would like to visit a restaurant where this dish is served	1–7 agreement
Post	Word-of-mouth intention	この料理について友人・知人に教えたい	I would like to tell friends or acquaintances about this dish	1–7 agreement

Instruction shown before the first dish: “You will now answer several questions about the first sturgeon-fritter dish. There are no correct answers; please report the impressions you personally formed.” The same wording was used for the second dish, replacing “first” with “second.” No sustainability or production-method information was included in these instructions.
